# Maternal Rumen Bacteriota Shapes the Offspring Rumen Bacteriota, Affecting the Development of Young Ruminants

**DOI:** 10.1128/spectrum.03590-22

**Published:** 2023-02-21

**Authors:** Shuwen Jin, Zhe Zhang, Gonghai Zhang, Bo He, Yilang Qin, Bin Yang, Zhongtang Yu, Jiakun Wang

**Affiliations:** a Institute of Dairy Science, College of Animal Sciences, Zhejiang University, Hangzhou, China; b MoE Key Laboratory of Molecular Animal Nutrition, Zhejiang University, Hangzhou, China; c Institute of Animal Breeding, College of Animal Sciences, Zhejiang University, Hangzhou, China; d Department of Animal Sciences, The Ohio State University, Columbus, Ohio, USA; Iowa State University

**Keywords:** dam, fermentation, growth traits, gut microbiome, lamb, rumen bacteriota, sheep

## Abstract

The maternal rumen microbiota can affect the infantile rumen microbiota and likely offspring growth, and some rumen microbes are heritable and are associated with host traits. However, little is known about the heritable microbes of the maternal rumen microbiota and their role in and effect on the growth of young ruminants. From analyzing the ruminal bacteriota from 128 Hu sheep dams and their 179 offspring lambs, we identified the potential heritable rumen bacteria and developed random forest prediction models to predict birth weight, weaning weight, and preweaning gain of the young ruminants using rumen bacteria as predictors. We showed that the dams tended to shape the bacteriota of the offspring. About 4.0% of the prevalent amplicon sequence variants (ASVs) of rumen bacteria were heritable (*h*^2^ > 0.2 and *P* < 0.05), and together they accounted for 4.8% and 31.5% of the rumen bacteria in relative abundance in the dams and the lambs, respectively. Heritable bacteria classified to *Prevotellaceae* appeared to play a key role in the rumen niche and contribute to rumen fermentation and the growth performance of lambs. Lamb growth traits could be successfully predicted using some maternal ASVs, and the accuracy of the predictive models was improved when some ASVs from both dams and their offspring were included.

**IMPORTANCE** Using a study design that enabled direct comparison of the rumen microbiota between sheep dams and their lambs, between littermates, and between sheep dams and lambs from other mothers, we identified the heritable subsets of rumen bacteriota in Hu sheep, some of which may play important roles in affecting the growth traits of young lambs. Some maternal rumen bacteria could help predict the growth traits of the young offspring, and they may assist in breeding of and selection for high-performance sheep.

## INTRODUCTION

The gut microbiota plays an important role in host nutrition and health. It has also been widely used in distinguishing some diseases (1), assessing and predicting disease risk (2), and evaluating the causal effects of its variations on health outcomes (3) in humans. The gut microbiota impacts host health starting at the early stage of life. During gestation, the maternal gut microbiota can stimulate fetal neurodevelopment by promoting thalamocortical axonogenesis, protecting offspring from food allergy through stimulating the development of fetal immune tolerance (4–6), and alleviating the effects of prenatal stress on offspring’s gut and hypothalamus, all of which have lasting effects (7). Mothers can exert a strong effect on the acquisition and development of their offspring’s microbiota directly by passing “inheritable” microbes to the offspring (referred to as vertical inheritance) and indirectly by determining the genotypes and phenotypes of the offspring, both of which make some maternal microbes “heritable” (8–10). One longitudinal study on the microbiota of 25 mother-infant pairs showed that the maternal microbes from multiple body sites could be vertically transferred to the infants (11). One study using human twins showed that some maternal microbes were indeed heritable, and most heritable microbes were species of the family *Christensenellaceae* (12). In animals, including domesticated ruminants, however, the effect of the maternal microbiota on that of offspring is largely unknown.

Ruminants are the most important source of milk, meat, wool, skin, etc. They rely on the microbiota (mostly bacteria) in their rumen to convert plant biomass polymers, which are often indigestible for humans and animals, to nutrients (primarily volatile fatty acids [VFA] and microbial protein) that they can utilize. The ruminal microbiota is diverse, complex, and dynamic, responding to dietary changes to a greater extent than the intestinal microbiota because it is exposed to ingested feed directly, rather than to residual food left after digestion and absorption by the host. Additionally, unlike the epithelium of the other gut segments, the rumen epithelium is covered by a keratin layer, not a mucosal layer. The rumen epithelium lacks mucin and antimicrobial peptides (AMPs), such as defensins, cathelicidins, and IgA (13). Conceivably, the heritability of rumen microbes and the effect of the maternal rumen microbiota on the infantile rumen microbiota and development are likely distinct from those of monogastric animals and other gut segments.

Some rumen microbes are strongly associated with animal performance, including feed efficiency (14), metabolism (15), disease (8, 16), and methane emissions (17). The early colonization of the rumen and the development of the rumen microbiota have attracted much research interest, and several recent studies have reported that some taxa of the rumen microbes are heritable (18–20). In one study that compared three breeds of beef cattle (18), 59 of the detected microbial taxa (56 bacterial and 3 archaeal) were heritable, and they were associated with host feed efficiency traits and rumen fermentation characteristics. Twenty-two operational taxonomic units (OTUs) of rumen bacteria showed measurable heritability in one study that examined the association between host genetics and the taxonomic and functional composition of the rumen microbiota of 47 Holstein-Friesian dairy cows (19). These heritable OTUs were more closely related phylogenetically among themselves than expected by chance, and their abundance was associated with rumen fermentation characteristics and host physiological traits. Another study using dairy cows identified a subset of 39 heritable rumen OTUs, and they formed some of the co-occurrence network hubs linking rumen microbiota structure to host genetics and phenotypes, including rumen and blood metabolites, milk production efficiency, and methane emissions (20). Although having identified heritable rumen microbes and their association with some animal traits, these studies have limitations. First, heritable microbes were inferred from comparisons among breeds or associating animal genotypes with rumen microbiota, not determined by comparing the rumen microbiota between dams and their calves. Second, animals from different mothers were used, and thus, the studies were confounded by the difference in host genetics (both mothers and offspring) and littermates.

We hypothesized that direct comparison of the rumen microbiota between mothers and their offspring, between littermates and young animals from different mothers, and between mothers and young animals from other mothers would improve the identification of heritable rumen microbes. In the current study, we tested the above hypothesis using Hu sheep and their lambs, which were fed the same diet and group raised as mother-lamb pairs. The objectives were to determine the potentially heritable rumen microbes, their potential roles, and the effect of the maternal ruminal bacteriota (we focus on the bacteria only) on the growth of young animals. We adopted the Hu sheep as the model because it is prolific and can produce multiple littermates, reducing the difference among the young (21). We also compared the rumen bacterial profiles between and within litters. This study provided new insights into how the maternal rumen bacteriota might affect the early colonization and development of the rumen bacteriota and the growth of young ruminants and may inform the development of new methods to predict or improve the growth of offspring by maternal microbes.

## RESULTS

### Profiles of the rumen bacteriota of sheep mothers and lambs.

A total of 11,042,149 quality-filtered amplicon sequences (BioProject number PRJNA874721) were obtained from 25,364,412 original reads for the rumen samples of 128 Hu sheep dams and their 179 offspring lambs, with an average of 35,967 ± 9,514 (mean ± standard deviation [SD]) sequences per sample. The sequencing depth coverage reached >99.8% on average (99.7% to 100% [see Fig. S1 in the supplemental material]). In total, 33,530 ASVs were identified. After removal of the rare or sparse ASVs (each detected in <5% of all the samples), 2,654 ASVs counted for 757,067 sequences (93.1% of total quality-filtered amplicon sequences) were retained and considered prevalent (Table S2), and they were used in the comparative bacteriota analyses between dams and lambs.

**FIG 1:**
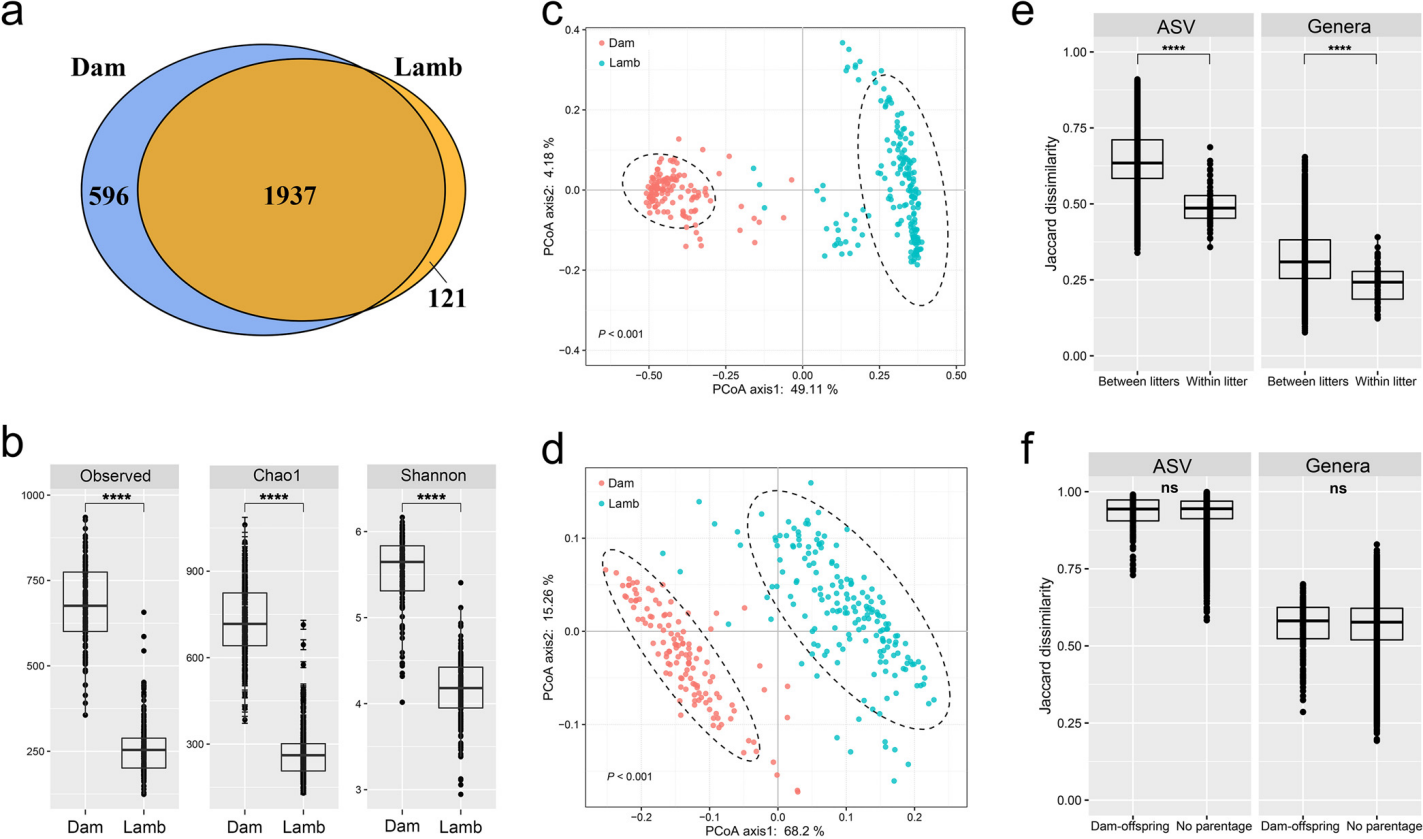
Comparison of the rumen bacteriota of 128 Hu sheep dams and 179 Hu sheep lambs. (a) Venn diagram showing the shared and unique ASVs; (b) box-and-whisker plots comparing the alpha diversity metrics calculated with all the ASVs; (c and d) principal-coordinate analysis (PCoA) plots based on Bray-Curtis dissimilarity and weighted UniFrac distance, respectively, at the ASV level, with significance tested using PERMANOVA (Adonis, permutation = 999, the area enclosed by the dashed lines represents the 95% confidence interval); (e) binary Jaccard dissimilarity of the rumen bacteriota of lambs within the same litters and between litters; (f) binary Jaccard dissimilarity of the rumen bacteriota between dams and their lambs and between dams and lambs from other mothers. Asterisks indicate a difference between groups based on the Wilcoxon test. ****, *P ≤ *0.0001. ns, no significance.

The bacteriota structure of dams was distinct from that of lambs. Among the 2,654 prevalent ASVs, 596 were exclusively found in dams, while 121 were exclusively found in lambs (Fig. 1a). The richness estimate (Chao1), evenness, and diversity index (Shannon) were all significantly higher (Wilcoxon test, *P < *0.0001) in dams than lambs (Fig. 1b). The principal-coordinate analysis (PCoA) plots based on Bray-Curtis dissimilarity (Fig. 1c) and weighted UniFrac dissimilarity (Fig. 1d) showed that the bacteriota of dams differed significantly from that of lambs (permutational multivariate analysis of variance [PERMANOVA] by Adonis, *P < *0.001). Binary Jaccard dissimilarity was calculated at both the ASV and genus levels. Based on Jaccard dissimilarity, littermates had a more similar rumen bacteriota than lambs from different litters at the ASV and genus levels (Wilcoxon test, Holm-adjusted *P* values of 3.00 × 10^−28^ and 4.70 × 10^−13^ at the ASV and genus levels, respectively) (Fig. 1e), while no difference in bacteriota was found between dams and their lambs or between dams and lambs from other mothers (Fig. 1f).

*Bacteroidales* and *Lachnospirales* were the most predominant orders in both dams and lambs, with *Bacteroidales* reaching average relative abundances of 72.6% in dams and 56% in lambs and *Lachnospirales* reaching average relative abundances of 12.3% in dams and 22.4% in lambs. *Prevotellaceae* (50.5% in dams and 53.0% in lambs) and *Lachnospiraceae* (12.1% in dams and 22.3% in lambs) were the most abundant families. The top 10 abundant genera included (in descending order) *Prevotella* (37.8% in dams versus 10.7% in lambs), *Prevotella_7* (2.4% versus 31.5%), *Lachnospiraceae* NK3A20 group (1.4% versus 5.3%), *Shuttleworthia* (0.5% versus 5.2%), *Prevotellaceae* UCG-001 (4.1% versus 1.8%), *Oribacterium* (0.7% versus 4.3%), *Rikenellaceae* RC9 gut group (4.6% versus 1.2%), *Ruminococcus* (1.3% versus 3.4%), *Prevotella_9* (0.3% versus 3.3%), and *Christensenellaceae* R-7 group (4.0% versus 0.5%) (Fig. S2).

### Some rumen bacteria are heritable.

**FIG 2:**
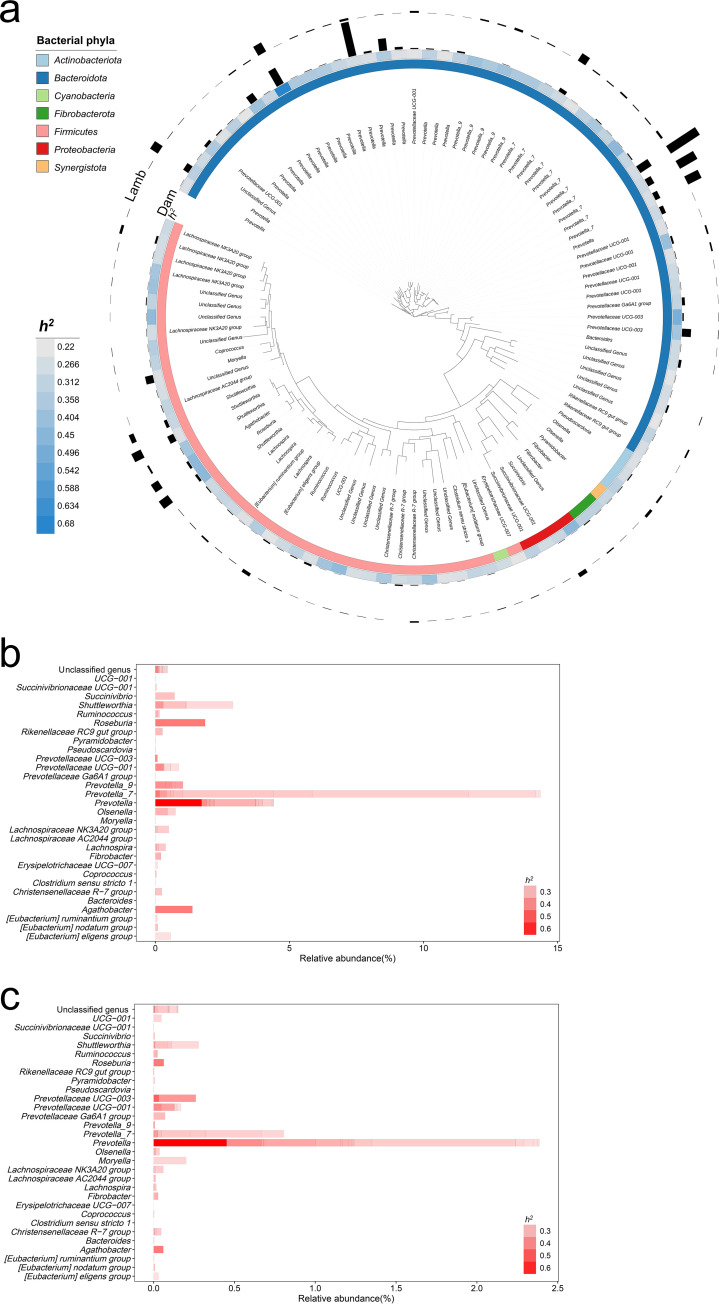
Heritable rumen bacterial taxa. (a) Phylogenetic tree of heritable ASVs (*h*^2^ > 0.2 and *P < *0.05). From the inner to the outer circles are shown the names of the genera, phyla to which the genera were assigned, the *h*^2^ values (as indicated by the heat map), and the relative abundance of the genera in dams and their offspring. (b and c) Relative abundance and distribution of the genera represented by heritable ASVs (*h*^2^ > 0.2 and *P < *0.05) in lambs and dams, respectively.

An ASV with a heritability value (*h*^2^) greater than 0.2 within a ≥95% confidence interval (22) and a *P* value less than 0.05 (23) was considered heritable. Based on this criterion, 106 ASVs (4.0% of the 2,654 prevalent ASVs) were identified to be likely heritable, and their heritability values (*h*^2^) ranged from 0.22 to 0.68. They were classified into seven phyla, 11 classes, 17 orders, 20 families, and 30 genera (Fig. 2a and Table S2). At the family level, 47 of the 106 (44.3%) heritable ASVs belonged to *Prevotellaceae*, followed by *Lachnospiraceae*, with 23 (21.7%) heritable ASVs. *Prevotella* had the most heritable ASVs (20), followed by *Prevotella_7* (11), and they were the top two genera with the highest relative abundance of heritable ASVs in both lambs (Fig. 2b and Table S2) and dams (Fig. 2c and Table S2). The heritable ASVs had combined relative abundances of 4.8% ± 3.84% in dams and 31.5% ± 10.86% in the lambs. Twelve of the 20 most abundant ASVs in the lambs were heritable, while only 2 of the 20 most abundant ASVs in the mothers were heritable. The top nine heritable ASVs had a heritability value above 0.4, and they included ASV10 (taxonomically assigned to *Prevotella*; *h*^2^ = 0.68), ASV593 (*Prevotellaceae* UCG-003; *h*^2^ = 0.49), ASV192 (*Prevotella_7*; *h*^2^ = 0.44), ASV2647 (unclassified *Magnetospirillaceae*; *h*^2^ = 0.44), ASV11 (*Roseburia*; *h*^2^ = 0.44), ASV20 (*Agathobacter*; *h*^2^ = 0.43), ASV3928 (unclassified *Lachnospiraceae*; *h*^2^ = 0.42), ASV682 (unclassified [*Eubacterium*] *coprostanoligenes* group; *h*^2^ = 0.42), and ASV100 (*Prevotellaceae* UCG-001; *h*^2^ = 0.41).

### Co-occurrence of the rumen bacterial ASVs.

**FIG 3:**
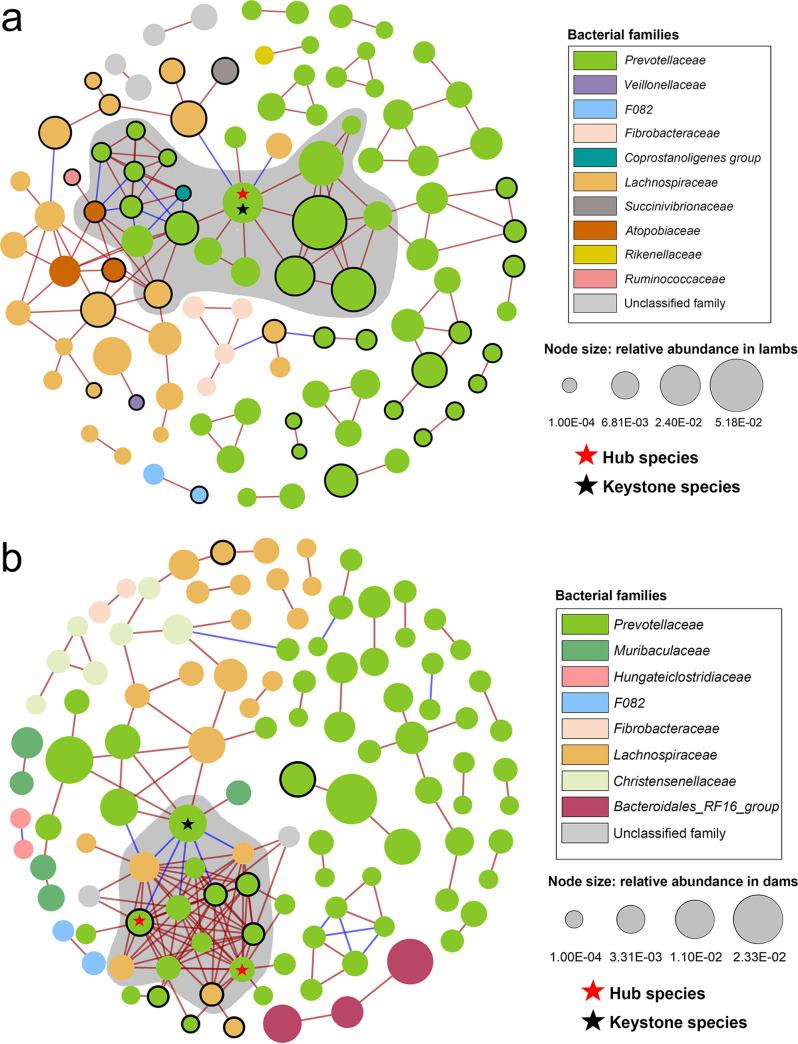
Co-occurrence networks of the rumen bacterial taxa of the lambs (a) and dams (b). Edges represent significant correlation (SparCC pseudo-*P* value < 0.01) with a SparCC value of greater than 0.5 or less than −0.5. Blue lines represent a negative correlation and red lines represent a positive correlation, with their thicknesses corresponding to the correlation coefficients. Nodes represent ASVs, and their sizes represent their relative abundances. The colors of the nodes indicate the families to which the ASVs were assigned. The nodes with a black outline represent heritable ASVs (*h*^2^ > 0.2 and *P < *0.05).

The co-occurrence network of rumen bacteria for the lambs (Fig. 3a) differed from that for the dams in both components and structures (Fig. 3b). The lamb network had a higher clustering coefficient, while the dam network had a higher average degree and network density (Table S3). The dam network had two hub species (nonheritable ASV4 and heritable ASV1, with an *h*^2^ of 0.26, both assigned to *Prevotella*) and one keystone species (ASV42, assigned to *Prevotella*), while the lamb network had ASV8 (unclassified *Prevotellaceae*) as both the hub and keystone species. Identified using molecular complex detection (MCODE) analysis (Fig. 3 and Table S4), the cluster with the highest MCODE network score included the hub ASVs, keystone ASVs, and their neighbors. For the dam network, the cluster with the highest MCODE network score (10) included 10 ASVs of *Prevotellaceae* and 4 ASVs of *Lachnospiraceae*. For the lamb network, the cluster with the highest MCODE network score (5) had 19 ASVs, including 16 ASVs of *Prevotellaceae*, and one ASV each of *Lachnospiraceae*, (*Eubacterium*) *coprostanoligenes* group, and *Atopobiaceae*. Five of the 14 clusters of ASVs in the dam network and 12 of the 19 clusters of ASVs in the lamb network were heritable (Table S4).

### Some rumen bacteria are associated with lamb growth traits and rumen fermentation characteristics.

**FIG 4:**
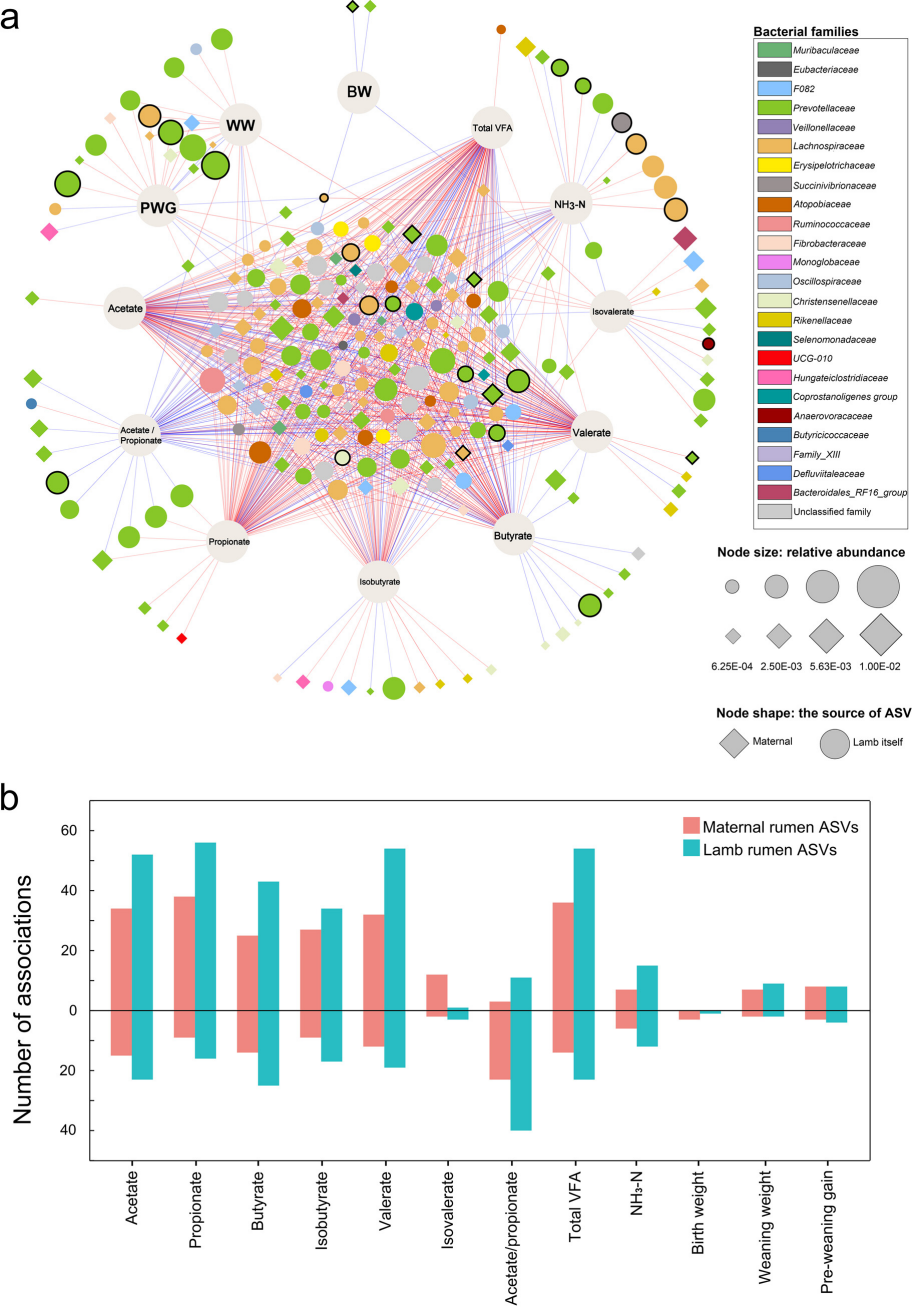
Associations between rumen bacterial ASVs and lamb growth traits and rumen fermentation characteristics. (a) Network of correlations between bacterial ASVs and growth traits and rumen fermentation characteristics of lambs. Edges represent significant Spearman collections (Benjamini-Hochberg-adjusted *P* value < 0. 05). Blue lines represent a negative correlation and red lines represent a positive correlation, with their thicknesses corresponding to the correlation coefficients. The size of each node represents their relative abundance, and the color of each node indicates the family to which the ASV was assigned. The nodes with a black outline represent heritable ASVs (*h*^2^ > 0.2 and *P < *0.05). (b) Numbers of ASVs that showed a positive correlation (bars above the zero line) or negative correlation (bars below the zero line) with one or more lamb growth traits or rumen fermentation characteristics.

In total, 118 ASVs of the lambs and 112 maternal ASVs showed a correlation with at least one of the ruminal fermentation parameters (including concentrations of NH_3_-N and individual VFAs) and/or one of the growth traits (birth weight [BW], weaning weight [WW], and preweaning weight gain [PWG]) of the lambs (Fig. 4a). The lamb rumen ASVs formed 337 positive correlations and 185 negative correlations with one of the traits, while the dam rumen ASVs formed 229 positive correlations and 112 negative correlations with one of the traits (Fig. 4b). Most of the correlations were between ASVs and ruminal fermentation parameters, and more ASVs from the lambs had correlations than the ASVs from their respective mothers, but more correlations between ASVs and isovalerate concentration were observed in dams. Only some ruminal ASVs were correlated with the growth traits of lambs (4 with BW, 20 with WW, and 23 with PWG). Among all the trait-associated ASVs, 20 from the lambs themselves and 6 from their mothers were heritable (Fig. 4a). They aggregated in *Prevotellaceae* and *Lachnospiraceae*, and one ASV each was assigned to *Christensenellaceae*, *Anaerovoracaceae*, and *Succinivibrionaceae* (Fig. 4a).

### The rumen bacteriota of lambs and their mothers can help copredict lamb growth traits.

**FIG 5:**
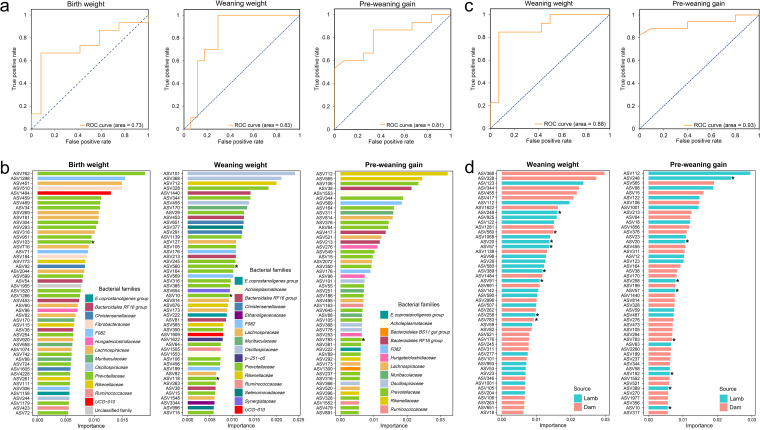
Prediction of birth weight, weaning weight, and preweaning gain using random forest models and the ASVs of lambs and their respective dams. (a) Receiver operating characteristic (ROC) plots of the random forest classification (RFC) for birth weight, weaning weight, and preweaning gain predicted using maternal ASVs; (b) the top 50 important maternal ASVs included in the predicting models of weaning weight and preweaning gain; (c) ROC plots of the RFC for weaning weight and preweaning gain predicted using rumen bacteriota of both lambs and dams; (d) the top 50 important ASVs included in the predicting models of weaning weight and preweaning gain. The ASVs with an asterisk represent heritable ASVs (*h*^2^ > 0.2 and *P < *0.05).

Because some ASVs (both of the lambs and of the respective mothers) correlated with one or more of the three lamb growth traits, we used the abundance of these ASVs to copredict growth trait groups (high versus low, corresponding to the top and the bottom 25% for each litter size) of the lambs (Table S1). The groups with high versus low BW, WW, and PWG were successfully predicted using the rumen ASV of dams (Fig. 5). The prediction accuracy was 0.71, 0.77, and 0.83 for predicting the high versus low groups of BW, WW, and PWG using maternal ASVs, respectively. The area under the receiver operating characteristic (ROC) curve, which describes the robustness of binary classification models, was 0.73, 0.83, and 0.81 for the high versus low groups of BW, WW, and PWG, respectively (Fig. 5).

When the rumen ASVs of both lambs and dams were included in the prediction model, the prediction accuracy increased to 0.80 and 0.87, and the area under the ROC curve increased to 0.88 and 0.93 for the high versus low groups of WW and PWG, respectively. In the random forest classifier (RFC) model predicting WW groups, 26 of the top 50 predictor ASVs with the highest importance scores were maternal with a combined importance score of 0.32, while the 24 lamb ASVs had a combined importance score of 0.28. In the model predicting PWG, 31 of the top 50 predictor ASVs with the highest importance scores were maternal and their importance scores added up to 0.27, while the remaining 19 lamb ASVs had a combined importance score of 0.22. Most of the top 50 important predictor ASVs belonged to the families *Prevotellaceae* and *Lachnospiraceae* in the models predicting the two WW and the two PWG groups (Fig. S3 and Table S5). Eight (2 maternal and 6 lamb) of the top 50 important predictor ASVs in the WW-predicting model were heritable, while 8 (1 maternal and 7 lamb) of the top 50 important predictor ASVs in the PWG-predicting model were heritable.

## DISCUSSION

Comparative analyses of the rumen microbiota among animals with different genotypes and association studies of rumen microbiota and host genetics suggest that some of the rumen bacteria are heritable (10, 18, 19). Leveraging multiple offspring, we provided new evidence of heritable rumen bacteria by comparing the rumen bacteriota between mother sheep and their lambs, among littermates, and among nonlittermates. Although some rumen microbes were suggested to be heritable and associated with host performance (18–20, 22), it is not known how the maternal rumen bacteriota may affect the growth of their offspring through these heritable taxa and if the growth traits of offspring can be predicted based on maternal rumen microbes. Bridging the above gaps, we identified the potential association between the maternal rumen bacteriota and the infantile rumen bacteriota by comparatively analyzing the ruminal bacteriota of both dams and lambs and used random forest machine learning in identifying rumen bacteria that could help predict the growth of the young ruminants.

Although maternal heredity can be considered deterministic, the vertical inheritance of rumen microbes is largely stochastic because it primarily depends on chance. In this experiment, four or five litters of lambs were group raised together with mothers before weaning and then separated from their mothers after weaning to allow for different scenarios of cross-inoculation that may contribute to the heritability of rumen bacteria. However, our results still showed that lambs from the same litter had more similar rumen bacteriota compared to those of different litters irrespective of proximity to dams, suggesting that host genetics could affect the heritability of some rumen bacteria in sheep. This is consistent with the observation of human twins who shared a more similar gut microbiota than nontwins (24, 25). The proportion of heritable bacteria is within the range observed in dairy cows (20, 26), where cows and newborn calves are separated immediately after birth. This is intriguing because the chance of vertical transmission and thus inheritance should be increased by keeping the mother and her offspring together. Future studies can help determine to what extent vertical transmission contributes to the heritability of rumen bacteria by keeping some mothers and lambs or calves together while separating some other mothers and their offspring immediately after birth.

In our study, the heritable ASVs aggregated in *Prevotellaceae* and *Lachnospiraceae*, the two most abundant families of rumen bacteria. These results suggest that abundant ASVs could be more likely heritable. Most species of these two families are generalists capable of utilizing a number of nutrients, including starch, hemicellulose, and protein, and thus, they predominate in the rumen ecosystem. Their metabolic versatility and ecological fitness may also be attributable to the numerous heritable ASVs found in these two families. The revelation of other fibrolytic families of bacteria represented by heritable ASVs is not surprising given that sheep and lambs, like dairy cows and beef cattle, consume plant-based diets. For the fibrolytic bacteria, similar heritability had been observed in the rumen of beef cattle, but with the low heritability estimates of *Bacterioidota* members, Li et al. suggested that the heritability of rumen bacteria was inversely correlated with their metabolic versatility and ecological fitness, and the bacteria with more metabolic versatility could be more likely affected by diet (18). However, in the three studies on dairy cows, heritable OTUs showed a high relative abundance, and *Prevotellaceae* and *Lachnospiraceae* were also the families that had the highest heritability values (19, 20, 26).

We found that more ASVs were correlated with ruminal fermentation parameters than with growth traits. That is not surprising given that ruminal fermentation parameters directly reflect the fermentation activities of rumen bacteria, while growth traits are profoundly affected, or determined to a large extent, also by the metabolism and physiology of animals and by the nutrient content of the milk. As shown in other studies (27), the adult rumen bacteriota has a much higher species richness and diversity. It also has a much higher functional redundancy (28). Those bacteriota features might explain the fewer correlations between ASVs and ruminal fermentation parameters observed in dams than in the lambs. The more correlations observed in lambs than in dams highlight the essential role of rumen bacteria in the early period of life and corroborate the importance of this early period to the assembly of the rumen microbiota when it is much less resilient and much more responsive than the adult rumen microbiota (29). As revealed in the correlation analysis, more correlating ASVs were heritable in lambs than in dams, highlighting the importance of rumen bacteria in determining young animal growth in a heritable manner.

Some maternal and offspring ruminal ASVs appeared to be correlated with one or more traits of their offspring. We thus evaluated whether it is feasible to use those maternal ASVs to potentially predict some growth traits of their offspring. As a bagging algorithm of machine learning, RFC integrates multiple decision trees into a single classifier, and it has been successfully used in constructing predictive models based on microbiota data (30, 31). In our study, the maternal RFC models could be used to predict BW, WW, and PWG of the offspring (Fig. 5a and b and Table S5), with a prediction accuracy reaching 0.83 for PWG. The growing demand for animal-derived protein by the expanding and more affluent human population necessitates efficient and sustainable livestock production. Ewe efficiency is defined as the ratio of the total WW of her litter and her live weight at breeding (32). Direct selection for reduced ewe mature size will, on average, reduce the size of offspring (33), which can have repercussions for the efficiency of the entire sheep industry. Therefore, for ewes with a similar live weight, a heavier WW indicates more efficient animals than a lighter WW, and thus, a higher PWG is more efficient than a lighter PWG. Because the composition and structure of the maternal rumen bacteriota could be predictive of the growth traits of their offspring, these predictor ASVs in the maternal RFC models, especially those that are heritable, can serve as biomarkers assisting the selection of ewes for higher efficiency before breeding. This can help save feeding and breeding cost of fertile ewes.

To evaluate the potential contribution of heritable taxa of rumen bacteria to the growth traits of young animals, we also constructed RFC models using the rumen bacteriota data of both lambs and their mothers (referred to as coprediction models). As in the maternal RFC models, predictor bacteria belonging to *Prevotellaceae* dominated the top 50 important ASVs, followed by *Lachnospiraceae* and F082 in the coprediction models for WW and PWG. The predictor bacteria belonging to these families came nearly equally from dams and their offspring, but none of the top 50 important predictor ASVs was shared between dams and lambs. This might be attributable to the much higher species richness, diversity, and functional redundancy of adult rumen bacteriota than that of lambs. Compared to the maternal RFC models, the coprediction models had more predicter ASVs that were heritable and mostly came from lambs. These heritable predictor ASVs aggregated in *Prevotella* and *Prevotella_7*, from either the dams or lambs. Different from some other species of *Bacteroides*, *Prevotella* is metabolically versatile, performing a variety of metabolic functions that can benefit other rumen bacteria and improve the overall rumen function (34). That might suggest that the establishment of the metabolic functions of the rumen ecosystem is critical to the growth and development of lambs, irrespective of their heritability. The more heritable predictor ASVs from lambs than dams highlight that the heritable subsets of rumen bacteria, especially those belonging to *Prevotella*, occupy a specific ecological niche within the rumen ecosystem in the early period of life, and *Prevotella* might be the most important genus from dams improving the growth traits of lambs. However, the potential correlation between heritable predictor ASVs and the nutrients and energy content of the diets, especially the mother’s milk, should be studied in the future to provide a plausible mechanism.

Ruminants can “select” specific species of rumen bacteria based on their genetics and other factors, especially the diet they consume (35, 36). *Prevotella* is considered the most important genus in examining the diet-driven continuous interplay between humans and the gut microbiota and their coevolution, as demonstrated in developed countries, where most people have lost some of the gut *Prevotella* species due to the lack of diverse plant-based foods (37). Although *Prevotella* can be highly impacted by diet, especially a fiber-rich diet, its abundance can vary among dairy cows fed the same diet (38), which suggests that the host can determine its persistence and prevalence. If future studies can explain the heritability of *Prevotella* in the context of different ruminant diets, new strategies can be developed to promote animal growth by exploring the ability of the host to select heritable rumen bacteria to purposefully improve the heritability of specific microbes, especially those of *Prevotella*, by changing diets.

More definitive analyses, such as microbial source tracking (e.g., SourceTracker) (39), coupled with a study design that separates some mothers and their newborn lambs immediately after birth, keeps other mothers and their newborns together until weaning, and samples the rumen microbiota longitudinally multiple times from birth to weaning, shall help discriminate vertically inheritable from heritable rumen microbes, validate their possible routes of transmission, and reveal their roles in determining growth traits of young animals in future studies. The assembly process and the eventual assemblage of the rumen microbiota are mainly determined by deterministic factors (e.g., host genotype, age, and diet), but stochastic forces (e.g., passive dispersal, diversification, and ecological drift) can have a significant impact (40). Differences in host genetics, age, and diet, together with random stochastic forces, may explain the difference in rumen/gut microbiota heritability. Future studies can help understand how these factors determine or affect the rumen microbiota of young ruminants by analyzing the abundance and dynamics of heritable rumen microbes at the strain level, which cannot be achieved by sequencing and analyzing one partial region of the 16S rRNA gene, as we did in the present study, or even the full length of the 16S rRNA genes. Genome-centric analyses of metagenome-assembled genomes can provide strain-level resolution and shall be used in future studies of heritable rumen microbes.

### Conclusion.

Comparatively analyzing the rumen bacteriota of mothers and their lambs using metataxonomics, we found that about 4.0% of the rumen bacteria were heritable in Hu sheep, and they were phylogenetically diverse, representing many bacterial taxa. More heritable bacteria are members of the predominant families *Prevotellaceae* and *Lachnospiraceae* than of other families. The heritability of rumen bacteria might be associated with or determined by their abundance and ecological fitness. The heritable ruminal bacteria dominated the early colonization of the rumen, and some of them appeared to contribute to growth performance. Some maternal rumen bacteria could help predict the growth traits of the young offspring and may assist in breeding selection.

## MATERIALS AND METHODS

### Animals and sample collection.

Hu sheep dams and their offspring were raised in a commercial sheep breeding farm from May to August 2019. All procedures involving animals were approved by the Animal Care Committee of Zhejiang University (Hangzhou, China). Hu sheep dams were group raised in wooden pens with a slotted floor under the exactly same conditions, with free access to water and bamboo shell silage. All the lambs stayed with mothers until weaning at 45 days of age and had free access to water and pellet feed. After weaning, the lambs were moved to another barn, separated into groups based on age, and kept in wooden pens with a slotted floor. Table S1 summarizes the information on dam-lamb pairs, rumen fermentation characteristics of dams and lambs, and the growth traits (birth weight [BW], weaning weight [WW], and preweaning weight gain [PWG]) of the lambs.

Rumen fluid samples were collected from the 128 Hu sheep dams (age = 1,032.7 ± 424.62 days, 59.7 ± 1.55 days after parturition) and their 179 offspring lambs 2 weeks after weaning (age = 59.8 ± 1.55 days). The rumen fluid samples were collected using oral stomach tubes before morning feeding. The first 20 mL of rumen fluid was discarded to avoid saliva contamination, and the oral stomach tubes were rinsed three times with a large syringe between sheep. One aliquot of each rumen fluid sample was snap-frozen in liquid nitrogen and subsequently stored at −80°C until DNA extraction. Another 4 mL of each rumen fluid sample was immediately acidified with 0.4 mL of 6-mol/L HCl and stored at −20°C until analyses for VFA using gas chromatography (GC-2010; Shimadzu Co., Ltd., Japan) (41) with isocaproic acid as the internal standard (42) and for NH_3_-N using a colorimetric method (43).

### Genomic DNA extraction and amplicon sequencing.

Metagenomic DNA was extracted from each rumen sample using the cetyltrimethylammonium bromide (CTAB) method (44), with minimal modifications. In brief, 1 mL of 2% CTAB solution was added to 0.2 to 0.3 g of each frozen rumen fluid sample and then homogenized using a bead-beating grinder (JXFSTPRP-48; Shanghaijingxin Experimental Technology, Shanghai, China) at 65 Hz for 90 s, with a 10-s break every 30 s. The nucleic acid pellet was dissolved in 50 μL of sterile water containing 0.1-mg/mL DNase-free RNase and then incubated at 37°C for 15 min (45). The concentrations of the DNA extracts were measured by a NanoDrop 2000 spectrophotometer (Thermo Scientific, USA), and the DNA quality was evaluated using agarose gel (1%) electrophoresis. The DNA solutions were stored at −20°C.

Individual amplicon libraries were prepared using PCR amplification of the V3-V4 hypervariable region of the 16S rRNA genes with primers 341F (5′-CCTAYGGGRBGCASCAG-3′) and 806R (5′-GGACTACNNGGGTATCTAAT-3′) (46), with each amplicon library having a unique barcode. The amplicon libraries were pooled at an equal molar ratio and paired-end (2 × 250) sequenced (47) on an Illumina NovaSeq 6000 system (Illumina, USA) by Novogene Bioinformatics Technology Co., Ltd. (Tianjin, China).

### Metataxonomic data processing and analysis.

After demultiplexing, paired-end sequence reads were analyzed using the DADA2 package (version 1.16 [http://benjjneb.github.io/dada2/tutorial.html]) in R (version 4.0.2) and its pipeline (48). Briefly, barcodes and primers were trimmed off. Dereplication was performed after the reads with N’s were filtered out. Denoising was performed using the divisive partitioning algorithm (49) with the default parameters. After the paired reads were merged and chimeras were filtered, one amplicon sequence variant (ASV) table was constructed. The ASVs were taxonomically assigned using the SILVA 16S reference data set (release 138.1) (50) with the assignTaxonomy function (51). The data files were then imported into the Phyloseq package (version 1.34.0) (52) as a Phyloseq object to remove archaeal and chloroplast sequences and to analyze the alpha and beta diversity, with each sample rarefied to 10,000 sequences. The ASV table was constructed after removing the ASVs that were detected in less than 5% of the samples (53). Alpha diversity metrics were compared using the Wilcoxon test. Principal-coordinate analysis (PCoA) was performed based on Bray-Curtis dissimilarity and weighted UniFrac dissimilarity created with the ASV table using the MicrobiotaProcess package (version 1.4.4) and visualized using the ggplot2 package (version 3.3.3). Statistical significance was tested using PERMANOVA (54) with 999 permutations.

Rank-based binary Jaccard dissimilarity was used to measure and compare the similarity of bacteriota among lambs from different mothers, among littermates, between the mothers and their lambs, and between mothers and lambs from other mothers with the vegan package (version 2.5.6). A maximum likelihood phylogenetic tree was constructed from representative ASV sequences after alignment with ClustalW using the default fasttree method of Qiime (55) and visualized using the online tool iTol (version 6.5.8 [https://itol.embl.de/]) (56). The relative abundance of each ASV was also calculated as the percentage of sequences of one ASV over the total sequences of each sample.

Microbial co-occurrence networks were constructed using the Python-based SparCC algorithm (57) with the ASVs that appeared in more than 20% of the dams or lambs as input data. The correlations with a SparCC correlation score greater than 0.5 or less than −0.5 and a *P* value less than 0.01 (bootstrap *n* = 100) were displayed using Cytoscape software (version 3.8.0) (58). The hub species (ASVs with the highest degrees) and keystone species (ASVs with the highest betweenness centrality scores) in the network were calculated using NetworkAnalyzer (59, 60), and the cluster with the highest network score was identified using MCODE with the default options (node score cutoff = 0.2, K-Core = 2, and maximum depth = 100) (61).

We evaluated the associations of individual prevalent ASVs (each found in 50% of the dams or lambs) with individual growth performance and rumen fermentation parameters of lambs using pairwise Spearman’s correlation with the *P* values corrected using the Benjamini-Hochberg (BH) method. The ASVs that showed a significant correlation (BH-corrected *P < *0.05) to a specific trait were considered ASVs that were associated with that trait. The correlation network was also visualized using Cytoscape software (version 3.8.0).

### Heritability estimation.

As done in a previous study (23), the heritability of each ASV was estimated using the following linear mixed model:

(1) y=Xb+Za+Wm+e

where *y* is the log-transformed relative abundance of an ASV; *b* is the vector of fixed effects, including year-season, group (dam or lamb), and age (days, as a covariate); *a* is the vector of additive genetic effects (breeding values), which is assumed to follow a zero-mean normal distribution $N\left(0,A\sigma_{a}^{2}\right)$, where $\sigma_{a}^{2}$ is the additive genetic variance and *A* is the numerator relationship matrix built from pedigree information; *m* is the vector of maternal effects and is assumed to be normally distributed with $N\left(0,I\sigma_{m}^{2}\right)$, where $\sigma_{m}^{2}$ is the variance of maternal effects and *I* is the identity matrix; *e* is the random residual vector following $N\left(0,I\sigma_{e}^{2}\right)$; and $\sigma_{e}^{2}$ is the random residual variance. *X*, *Z*, and *W* are incidence matrices related to the fixed and random effects in the model. The variance components were estimated using the AI-REML algorithm implemented in DMU software (62, 63). Based on the estimated variance components, the heritability of each ASV was defined as follows:

(2) $$h^{2}=\frac{\sigma_{a}^{2}}{\sigma_{a}^{2}+\sigma_{m}^{2}+\sigma_{e}^{2}}$$

The standard errors of heritability estimates were calculated according to an expansion of the Taylor series, and the *P* values of heritability were calculated using the unpaired two-tailed Student *t* test (23).

### Prediction of growth traits using the metataxonomic data.

A random forest classifier (RFC) model was built to predict the growth phenotypes of the lambs with the metataxonomic data. In detail, the lambs were ranked based on BW, WW, or PWG (calculated as the difference between WW and BW) for each litter size (1, 2, 3, or 4), and the top 25% and the bottom 25% of the lambs for each of the three traits were designated high (high_BW, high_WW, and high_PWG) or low (low_BW, low_WW, and low_PWG) groups as done in a previous study (64). The trait values of the high groups were significantly (*P <* 0.001) greater than those of the low groups (Table S1). The RandomForestClassifier function was used to predict the traits with the machine learning approach implemented in the Sklearn package (version 0.23.2 [https://scikit-learn.org/stable/]) in Python. The log-transformed relative abundance of individual bacterial ASVs from each lamb and its mother was used as the input data of the model. The importance of ASVs was ranked and selected by the feature_selection function (threshold = 1e−3). The n_estimators index was defined by the learning curves of 7-fold cross-validations, and the maximum depth and max_features indexes of the RFC were defined by the param_grid function to improve the model accuracy and prevent overfitting. The accuracy of RFC model was evaluated with 7-fold cross-validations, and the robustness of the RFC model was estimated with the receiver operating characteristic (ROC) curve, with 70% of the samples being used as the training data set and the remaining 30% as the test data set.

### Ethics approval.

All experimental protocols used in the current study were approved by the Animal Care and Use Committee of Zhejiang University (protocol number 17399), and all experimental procedures were performed following the approved protocols.

### Data availability.

The raw sequencing data generated in this study are publicly available in NCBI Sequence Read Archive under BioProject number PRJNA874721.
