# Supplementary material for: Maternal Rumen Bacteriota Shapes the Offspring Rumen Bacteriota, Affecting the Development of Young Ruminants
Source: Microbiol Spectr. 2023 Feb 21;11(2):e03590-22. doi: 10.1128/spectrum.03590-22 (PMC10100811; doi:10.1128/spectrum.03590-22)
Supplement: Supplemental file 2 — Fig. S1-3. Download spectrum.03590-22-s0006.pdf, PDF file, 1.9 MB [file spectrum.03590-22-s0006.pdf]

Additional Figures:

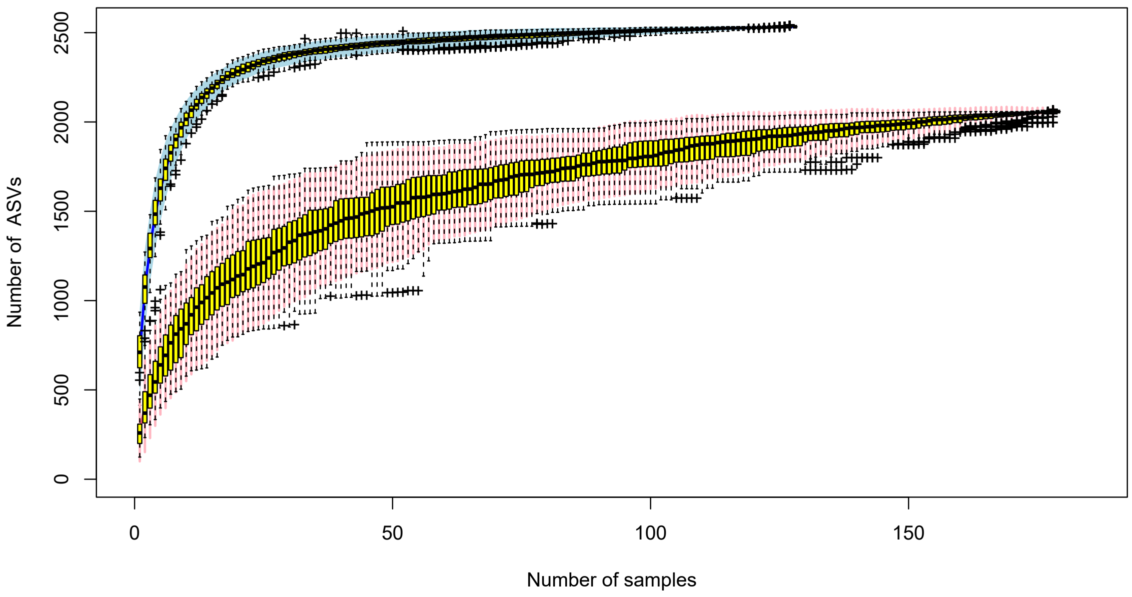

Fig. S1. Species accumulation boxplots of the rumen bacteriota of the Hu sheep dams (blue, n = 128) and lambs (n = 179, pink).

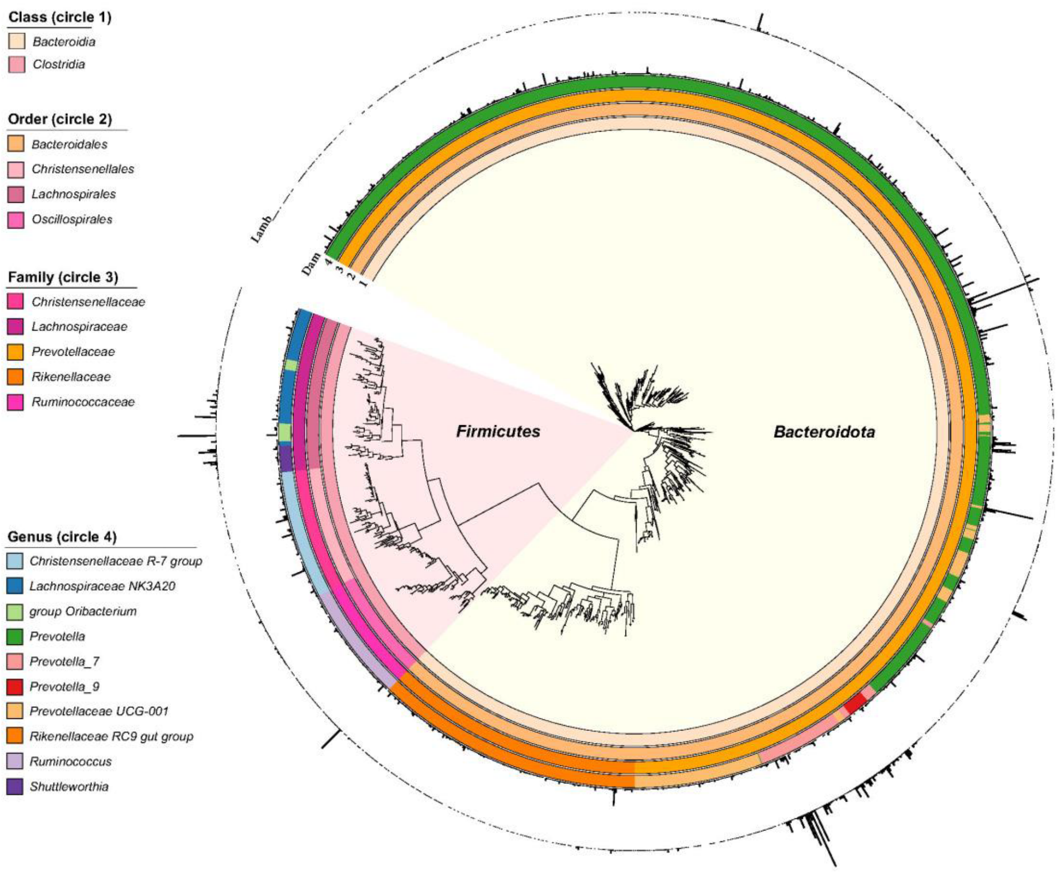

Fig. S2. A phylogenetic tree of the ASVs representing the top 10 most abundant genera. From the inner to the outer circles: classes, orders, families, genera, the relative abundance of the ASVs in dams and in lambs.

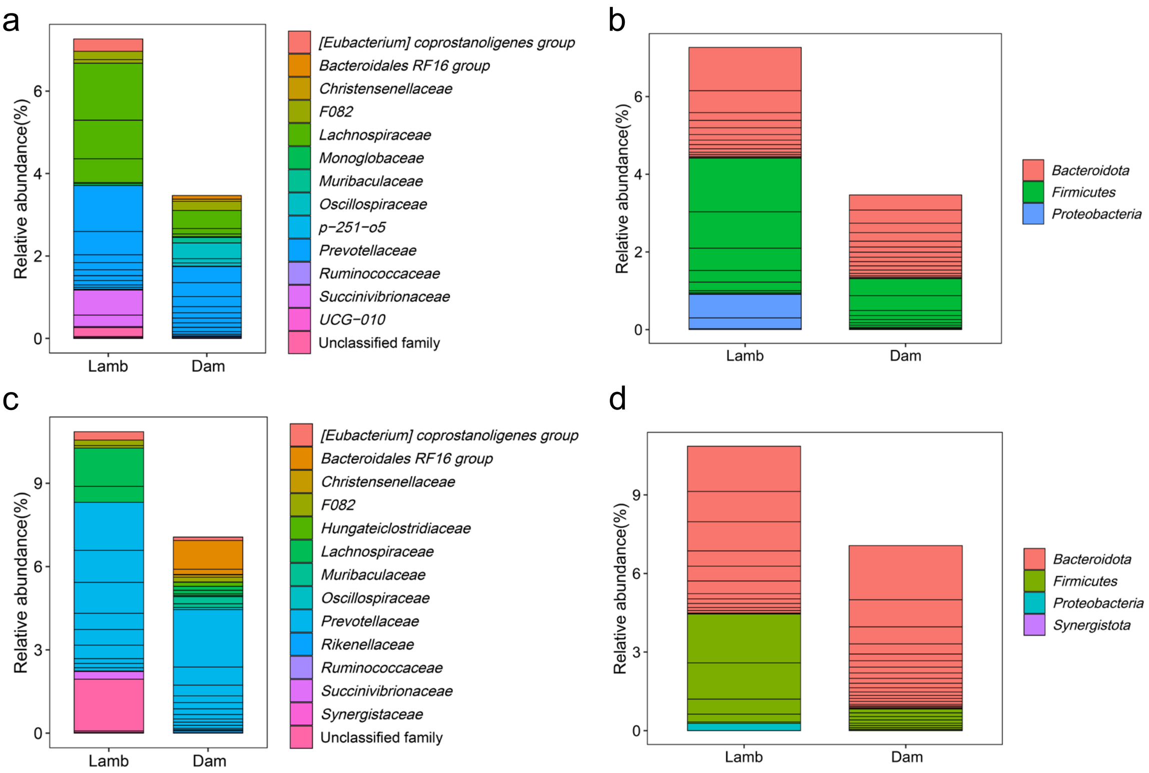

Fig. S3. Stacked histograms showing the relative abundance of the top 50 important ASVs in the RFC models. **a** and **b,** the families and phyla, respectively, represented by the top 50 important ASVs in the RFC model predicting weaning. **c** and **d,** the families and phyla, respectively, represented by the top 50 important ASVs in the RFC model predicting pre-weaning gain. Each cell represents one ASV.
